# CT and MRI of head and neck cutaneous lesions

**DOI:** 10.1259/bjro.20230006

**Published:** 2023-06-13

**Authors:** Jason Gan, Romman Nourzaie, Brent J. Doolan, Steve Connor

**Affiliations:** 1 Guys and St Thomas Hospital, London, United Kingdom; 2 King's College Hospital, London, United Kingdom

## Abstract

Cutaneous lesions are derived from the epidermis, dermis and cutaneous appendages. Whilst imaging may occasionally be performed to evaluate such lesions, they may be undiagnosed and demonstrated for the first time on head and neck imaging studies. Although usually amenable to clinical examination and biopsy, CT or MRI studies may also demonstrate characteristic imaging features which aid the radiological differential diagnosis. In addition, imaging studies define the extent and staging of malignant lesions, as well as the complications of benign lesions. It is important for the radiologist to understanding the clinical significance and associations of these cutaneous conditions. This pictorial review will describe and depict the imaging appearances of benign, malignant, overgrowth, blistering, appendage and syndromic cutaneous lesions. An increasing awareness of the imaging characteristics of cutaneous lesions and related conditions will help the framing of a clinically relevant report.

The skin is the largest organ of the human body and has a complex multilayered structure. Cutaneous and cutaneous appendages lesions are diverse, but since they are usually amenable to clinical examination and biopsy, they have received little attention in the imaging literature. CT or MRI studies of the head and neck may be used to delineate and stage skin tumours but may also demonstrate previously undiagnosed lesions. It is therefore important for the radiologist to understand the relevant imaging features, associations and significance of these cutaneous conditions.

The skin comprises of the epidermis and dermis (Figure 1). The epidermis consists of keratinocytes with melanocytes in its basal layer whilst the dermal layer is composed of fibroelastic connective tissue appendage structures such as hair follicles, sweat glands and sebaceous glands which traverse both layers. Cutaneous lesions frequently extend into the subcutaneous fat layer which lies immediately deep to the dermis. CT and MRI may demonstrate normal skin as a thin line of intermediate density and signal, and cutaneous lesions are usually manifest as a discrete mass or diffuse skin thickening.

**Figure 1.**
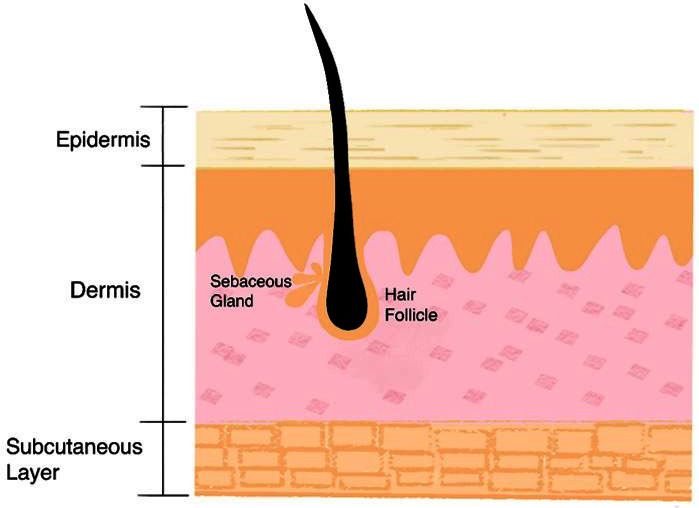
Diagram showing normal skin anatomy composed of the epidermis and dermis, and including sebaceous glands, hair follicles and sweat glands.

## Overgrowth

### Cutis verticis gyrata (CVG)

**Figure 2.**
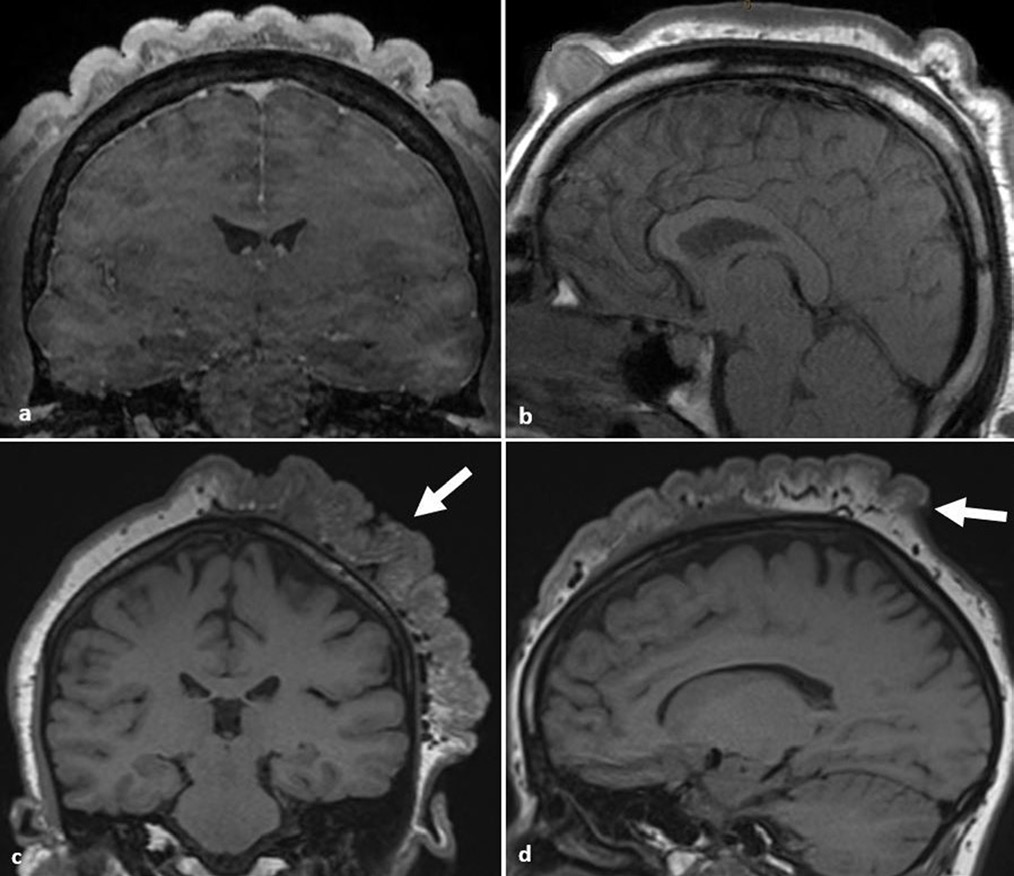
Cutis verticis gyrata. (**a, b**) Coronal and sagittal *T*_1_W contrast images demonstrate ridges and furrows of the dermis and subcutis of the scalp due to cutis verticis gyrata in a patient with acromegaly. Longitudinal ridges are best appreciated on the coronal image whilst transverse ridges are demonstrated on the sagittal image. (**c, d**) Coronal and sagittal *T*_1_W images demonstrating localised and unilateral ridges and furrows (arrows) secondary to cerebriform intradermal naevus (courtesy of Dr P. Touska).

CVG is a rare condition resulting in skin overgrowth on the scalp resulting in ridges and furrows resembling the gyriform appearance of the brain (Figure 2).^1^ It may be primary or secondary to other conditions (*e.g.* endocrine or dermatological).

Imaging usually demonstrates symmetric longitudinal ridges but they may be asymmetric or localised in secondary disease. Radiologists should interrogate the pituitary fossa and adjacent scalp for an underlying tumour.^1^

### Giant keloid

**Figure 3.**
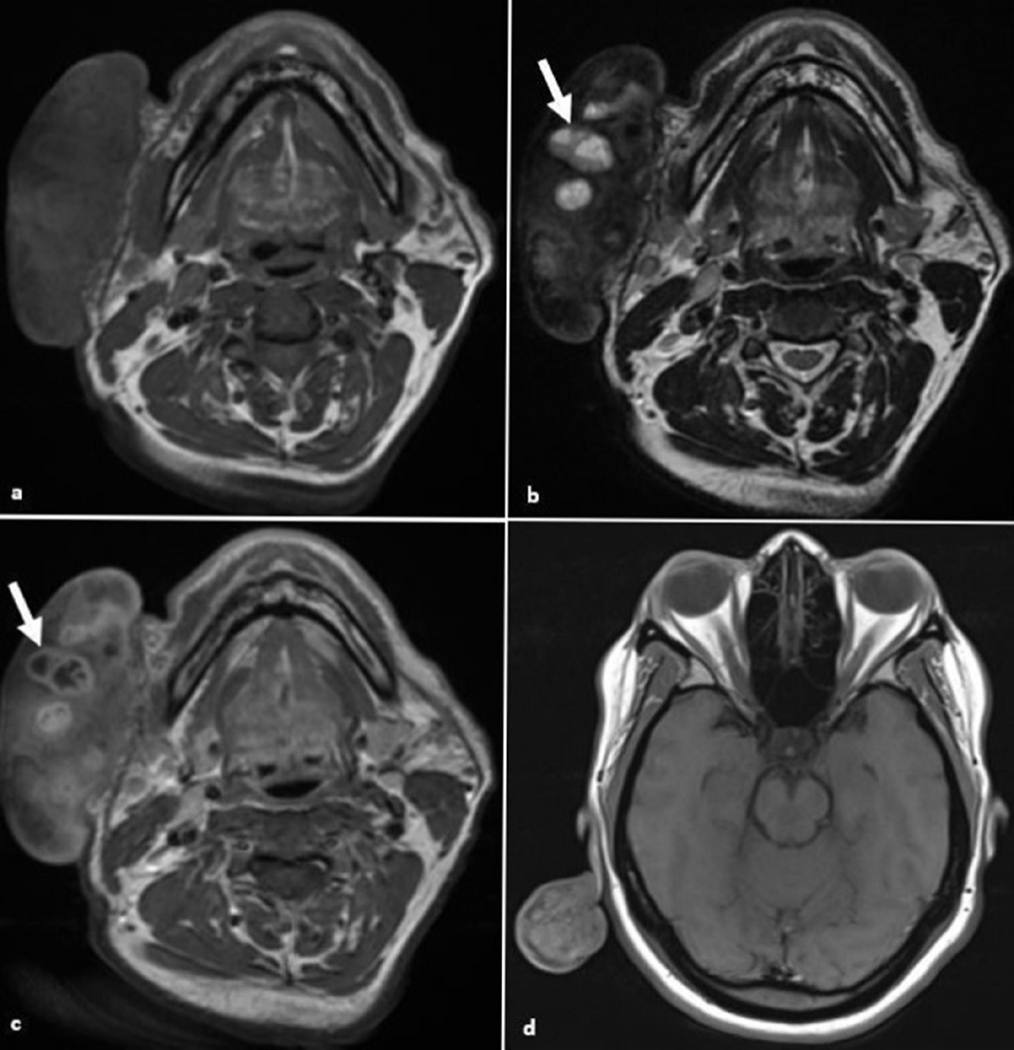
Giant keloid. (**a, b**) Axial *T*_1_W and *T*_2_W images demonstrate a large exophytic lesion arising from the right buccal cutis. It is of mixed signal with areas of non-enhancing T2 hyperintense necrosis (arrow). (**c**) There are areas of avid enhancement within the giant keloid (arrow). (**d**) Axial *T*_1_W image of a further keloid extending from the helix of the pinna.

A keloid is a benign disorganised fibroproliferative cutaneous growth in response to trauma or inflammation of the skin, which extends beyond the site of the original injury.^2^ Keloids are generally T2 hypointense. Enhancement is variable and the degree of vascularity may indicate proliferative activity (Figure 3). It is important to assess the thickness of the keloid as well as the deep extent.

## Benign skin lesions

### Haemangioma

**Figure 4.**
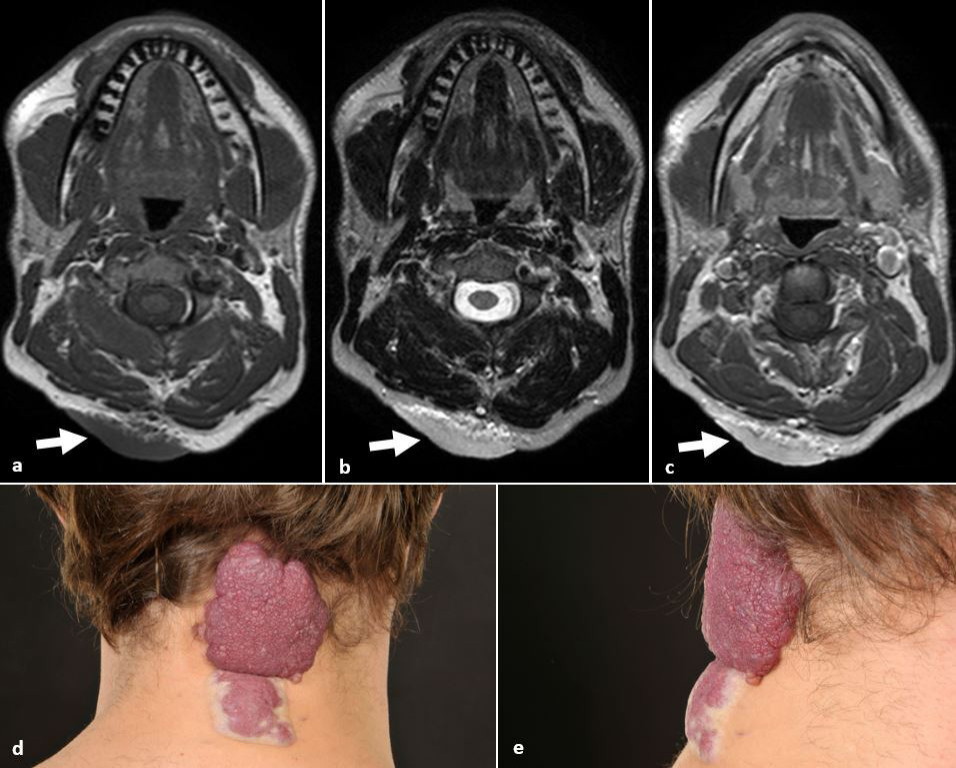
Haemangioma (**a, b**) Axial T1 images demonstrate an intermediate low/intermediate *T*_1_W signal and high *T*_2_W signal cutaneous and subcutaneous lesion in the posterior cervical soft tissues (arrow). (**c**) Axial T1 post-contrast image demonstrates homogeneous avid enhancement (arrow). (**c**) Axial T2 image demonstrates homogeneous avid enhancement. Some lesions show prominent flow voids. (**d, e**) Corresponding clinical photography in same patient demonstrating the erythematous/violaceous multilobulated thickened lesion of the dorsal neck resembling a “strawberry” due to the immature blood vessels. The inferior pole shows early white discolouration, suggestive of impending ulceration.

Haemangioma is a benign vascular tumour consisting of a hamartomatous growth of capillaries (Figure 4). They are the most common head and neck tumours of infancy. Whilst they grow rapidly in the early months, the majority of infantile haemangiomas stop growing by 10 months. Most have involuted by 5–7 years of age, however some subtypes persist.^3^ They may be associated with systemic hemangiomas or PHACES syndrome (posterior fossa malformations, haemangiomas, cerebral/cervical artery malformations, cardiac abnormalities/aortic coarctation, eye abnormalities).

Diagnosis is based on clinical assessment with imaging being potentially useful in cases of diagnostic uncertainty or to prompt treatment, when deep structures (*e.g.* orbit) are affected. Differential diagnosis includes congenital vascular malformations, and more aggressive vascular tumours such as Kaposiform haemangioendothelioma or angiosarcoma.^3^

## Skin appendage lesions

Skin appendage lesions may derive from the cutaneous layer but may extend into the subcutaneous fat layer on imaging.

### Epidermal inclusion cysts and pilar cysts

**Figure 5.**
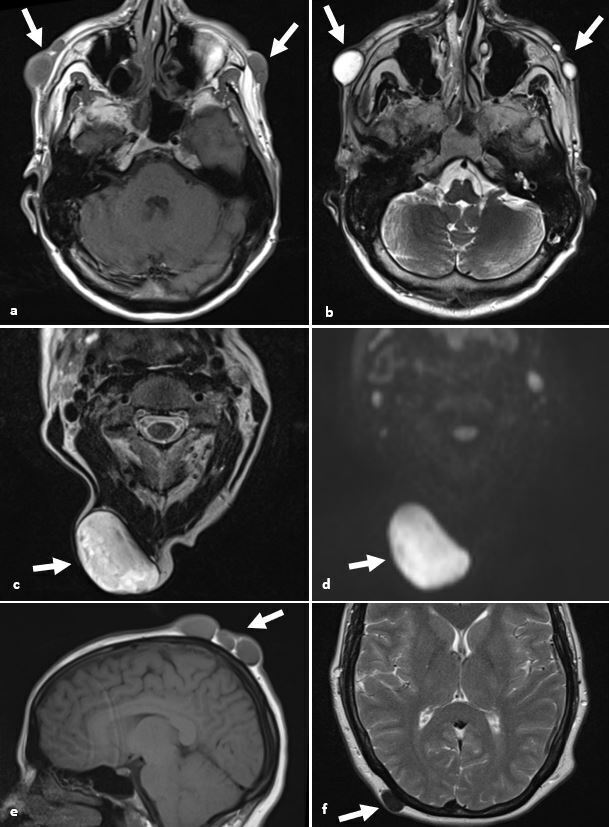
Epidermal inclusion cysts and pilar cysts. (**a, b**) Axial *T*_1_W and *T*_2_W images demonstrate well circumscribed T1 hypointense and T2 hyperintense dermal and subcutaneous lesions of the face (arrows) in keeping with fluid filled epidermal inclusion cysts. (**c, d**) Axial *T*_2_W and DWI images of a different patient demonstrate a heterogeneous T2 hyperintense lesion with restricted diffusion in keeping with a giant sebaceous cyst (arrows). (**e, f**) Sagittal *T*_1_W and axial *T*_2_W images of a further patient demonstrates T1 isointense and T2 hypointense lesions in keeping with pilar cysts (arrows). DWI, diffusion-weighted imaging.

Epidermal inclusion cysts (misnomer sebaceous cyst) are common cutaneous lesions. They are usually well-defined oval or spherical masses which demonstrate fluid density on CT, whilst T1 hypointensity, T2 hyperintensity and increased diffusion-weighted imaging (DWI) signal on MRI (Figure 5a–d), however the imaging characteristics are influenced by the variable composition of keratin, lipid containing debris and calcifications.^4^ A closely related lesion is the pilar cyst (trichilemmal cyst) which are keratin-filled lesions (Figure 5e and f) derived from the hair follicle.

### Hydradenitis suppurativa

**Figure 6.**
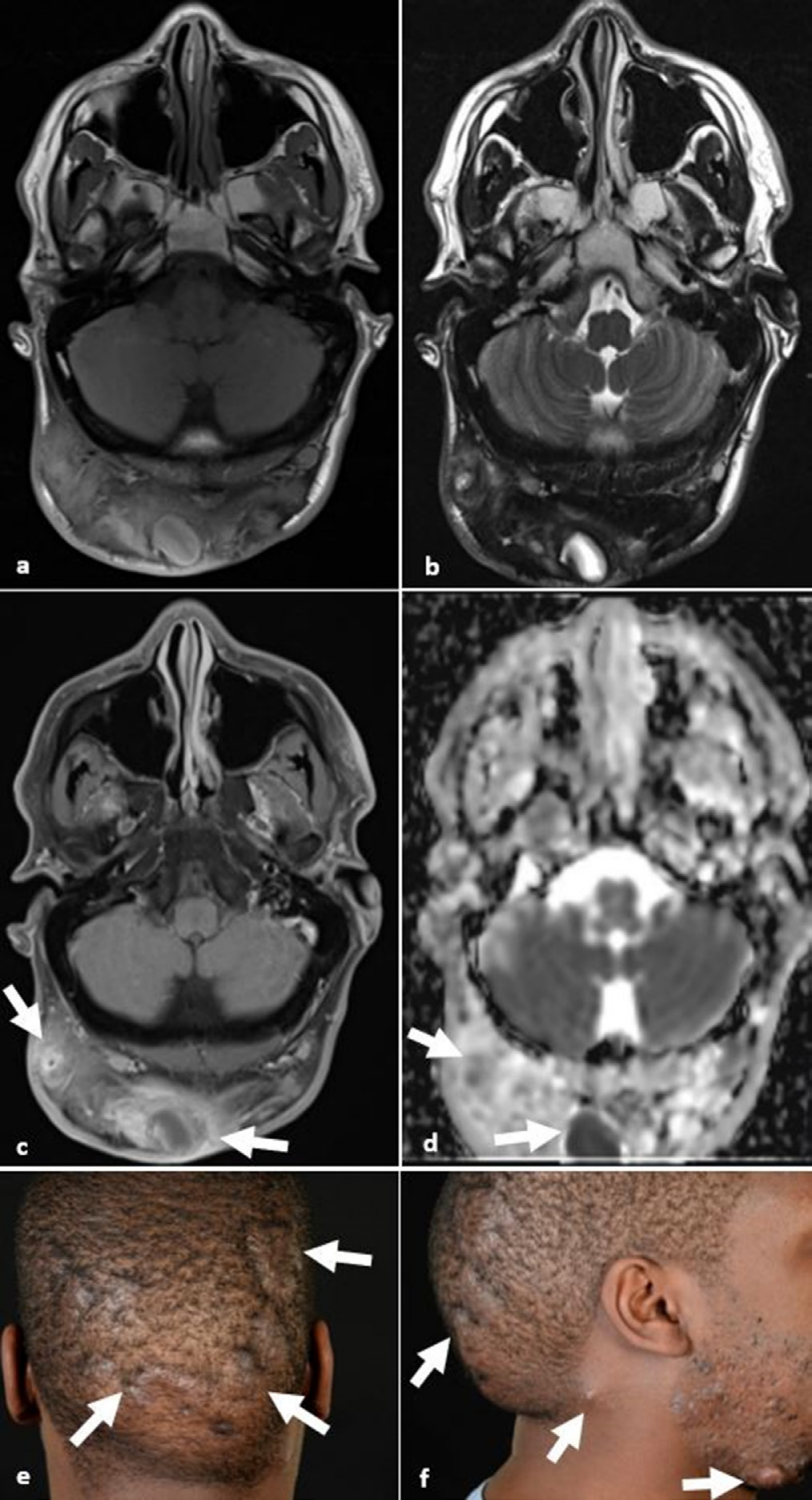
Hydradenitis suppurativa. (**a, b**) Axial *T*_1_W and *T*_2_W images demonstrate T1 hypo- to isointense and T2 hypointense signal within the occipital cutis with extensive deep extension to the subcutis. (**c, d**) Axial *T*_1_W post-gadolinium image and ADC map demonstrate peripheral enhancement (arrows in c) and restricted diffusion (arrows in d) in keeping with abscesses. (**e, f**) Clinical photos of the same patient demonstrating extensive involvement of the scalp, nape of the neck and lower facial involvement with nodules, abscesses and thick bands of scar tissue. ADC, apparent diffusion coefficient.

Hydradenitis suppurative is a chronic purulent condition of unknown aetiology. It is typically located in areas with apocrine follicles such as the axillae and groin, however, may occur in the head and neck. It is characterised by thickened skin, subcutaneous oedema, multiple abscesses and fistulae.^5^ The diagnosis is clinical but short tau inversion recovery sequences may be useful to evaluate the deep extent of abnormality and delineate abscesses (Figure 6). Differential diagnoses include lymphadenitis, carbuncles, and infected sebaceous cysts.

### Pilomatricoma

**Figure 7.**
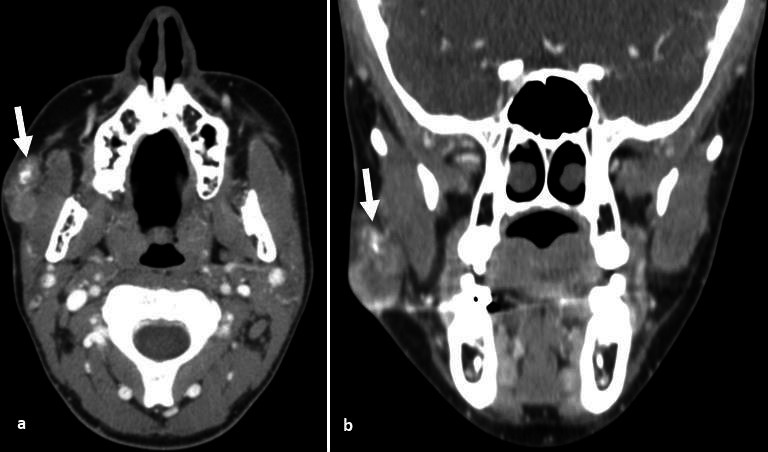
Pilomatricoma. Axial and coronal CT post-contrast demonstrates a lobulated, well-circumscribed buccal cutis and subcutis mass, superficial to the right masseter with linear foci of calcification (arrows) and mild enhancement.

Pilomatricoma is a benign tumour arising from the hair cell matrix, which frequently occurin the head and neck,and most commonly the mid face.^6^ They may be associated with other conditions (*e.g.* sarcoid) and syndromes. CT demonstrates well-circumscribed superficial enhancing masses with calcification (>80%) and variable enhancement (Figure 7). MRI shows characteristic bands of *T*_2_W hyperintense signal radiating from a low signal centre.^6^ The imaging differential diagnosis includes epidermal cysts, ossifying haematoma and foreign body reaction.

### Cylindroma

**Figure 8.**
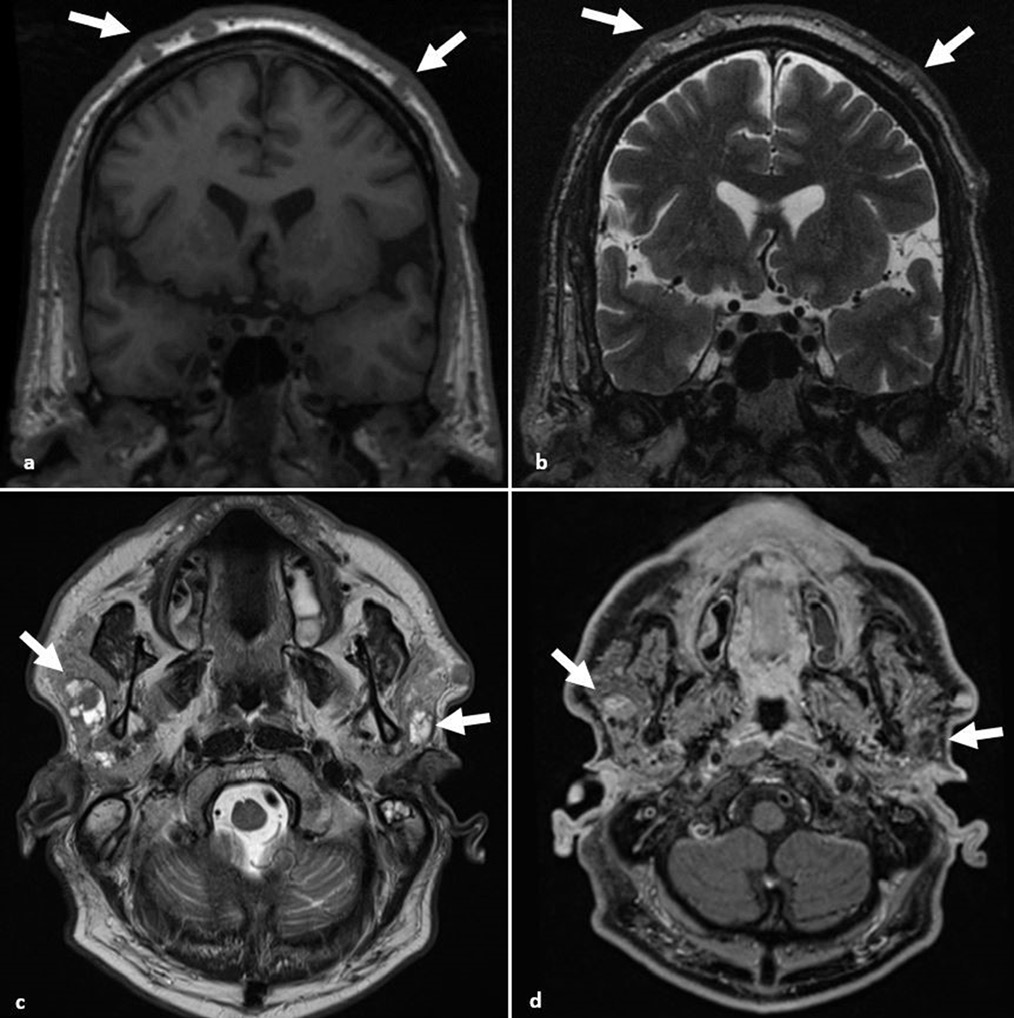
Cylindroma. (**a, b**) Coronal T1 and T2 images demonstrate multiple scalp soft tissue nodules with T1 hypointensity and T2 isointensity in keeping with cylindromas (arrows). (**c, d**) Axial *T*_2_W and *T*_1_W post-gadolinium images demonstrate T2 hyperintense non-enhancing parotid lesions (arrows), which are features of Brooke-Spiegler syndrome.

Cylindromas are appendage tumours of uncertain histogenesis but with a folliculosebaceous distribution. Whilst usually small, there may be large coalescing (turban tumours) of the scalp and forehead. Multiple cylindromas occur in Brooke-Spiegler syndrome, where multiple scalp nodules may be demonstrated on imaging (Figure 8).^7^ Other diagnostic considerations with multiple scalp lesions include pilar cysts or other syndromic scalp tumours (*e.g.* NF1) and hamartomas (Cowdens).

### Nodular hidradenoma

**Figure 9.**
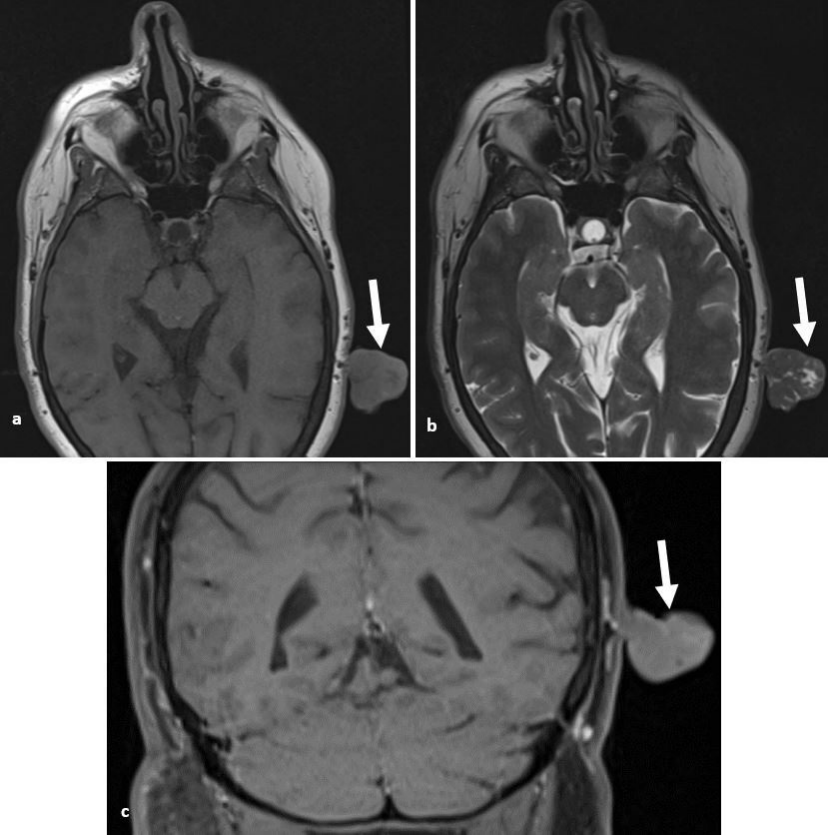
Nodular hidradenoma. (**a, b**) Axial *T*_1_W and *T*_2_W images demonstrates T1 hypointensity and T2 heterogeneity in an exophytic polypoid lesion arising from the cutaneous tissues of the left temporoparietal scalp (arrows). (**c**) Coronal *T*_1_W post-gadolinium fat sat coronal image demonstrates homogeneous mild enhancement of the lesion (arrow).

Nodular hidradenoma is a benign dermal-based skin appendageal neoplasm most commonly occurring in the scalp and face. It presents as a slow growing solitary solid or cystic lesion, with no overlying connection to the epidermis. Imaging appearances are non-specific with CT and MRI demonstrating solid enhancing and cystic components (Figure 9) with possible calcification.^8^

## Malignant

Malignant cutaneous lesions will be diagnosed clinically and by biopsy. Imaging may be used to evaluate the depth of the carcinoma, adjacent structures involvement, metastatic involvement and recurrent lesions.

### Cutaneous squamous cell carcinoma (SCC)

**Figure 10.**
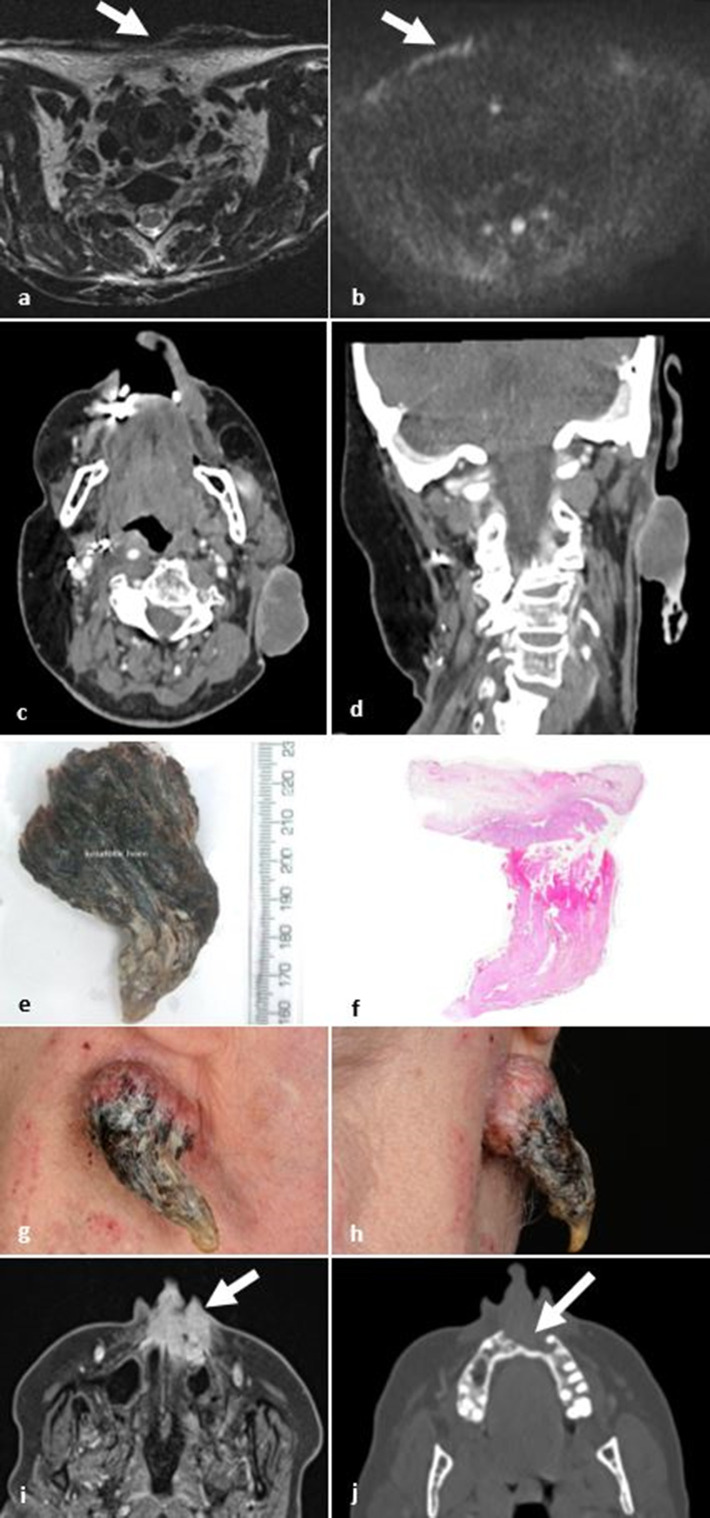
Cutaneous SCC. (**a, b**) Axial *T*_2_W and DWI images at the thoracic inlet. Diffuse T2 hypointense cutaneous and subcutaneous scar due to epidermolysis bullosa over the left inferior neck. Over the anterior lower neck, there is ulceration and increased DWI signal at the site of a complicating SCC (white arrows). (**c, d**) Axial and coronal post-contrast CT images demonstrates an exophytic mass arising from the left infra-auricular skin with central hypoattenuation and peripheral hyperdensity. At its inferior surface, there is a high attenuation horn-like projection in keeping with keratin. (**e**) Photo of detached keratin horn. (**f**) Composite wholemount H&E-stained section through keratin horn and underlying skin with exophytic SCC (courtesy of Dr Ann Sandison). (**g, h**) Clinical photos of the same patient demonstrating well-circumscribed, pink nodule underlying a hard conical projection of keratin. (**i, j**) T1 axial fat-saturated post-gadolinium and axial CT on bone windows demonstrating a cutaneous SCC arising within the nasolabial soft tissues (fig i arrow) and extending deeply to erode the anterior maxillary alveolus (fig j arrow). DWI, diffusion-weighted imaging; SCC, squamous cell carcinoma.

SCC represents 15–20% of skin cancers and occur on the sun exposed head and neck. Imaging may demonstrate cutaneous thickening or focal mass. The deep aspect may invade tissues whilst the superficial aspect may ulcerate (Figure 10a and b).^9^ Occasionally, there may be a primarily exophytic lesion with a keratin horn (Figure 10c–f) which mimics a benign keratosis. It is important to evaluate for imaging evidence of perineural spread and bony erosion which is critical for staging and treatment planning (Figure 10 i and j). Lymph node metastases in parotid and periparotid lymph nodes should be considered for cutaneous SCC originating in face, forehead, coronal scalp, periauricular area, and upper neck, whilst suboccipital or level five lymph node metastases may spread from posterior scalp and posterior neck primaries. SCC Tstaging (TNM8) is seen in Table 1.

**Table 1.** SCC T staging (TNM 8)

**Stage**	Primary Tumour
0	Tis = Carcinoma in situ
I	T1 - Tumour 2 cm or less
II	T2 = Tumour >2 cm but <4 cm
III	T3 = Tumour >4 cm or minor bone erosion or perineural invasion or deep invasion
IV	T4 = Tumour with gross cortical bone/marrow, skull base invasion and/or skull base foramen invasion

### Malignant melanoma (MM)

**Figure 11.**
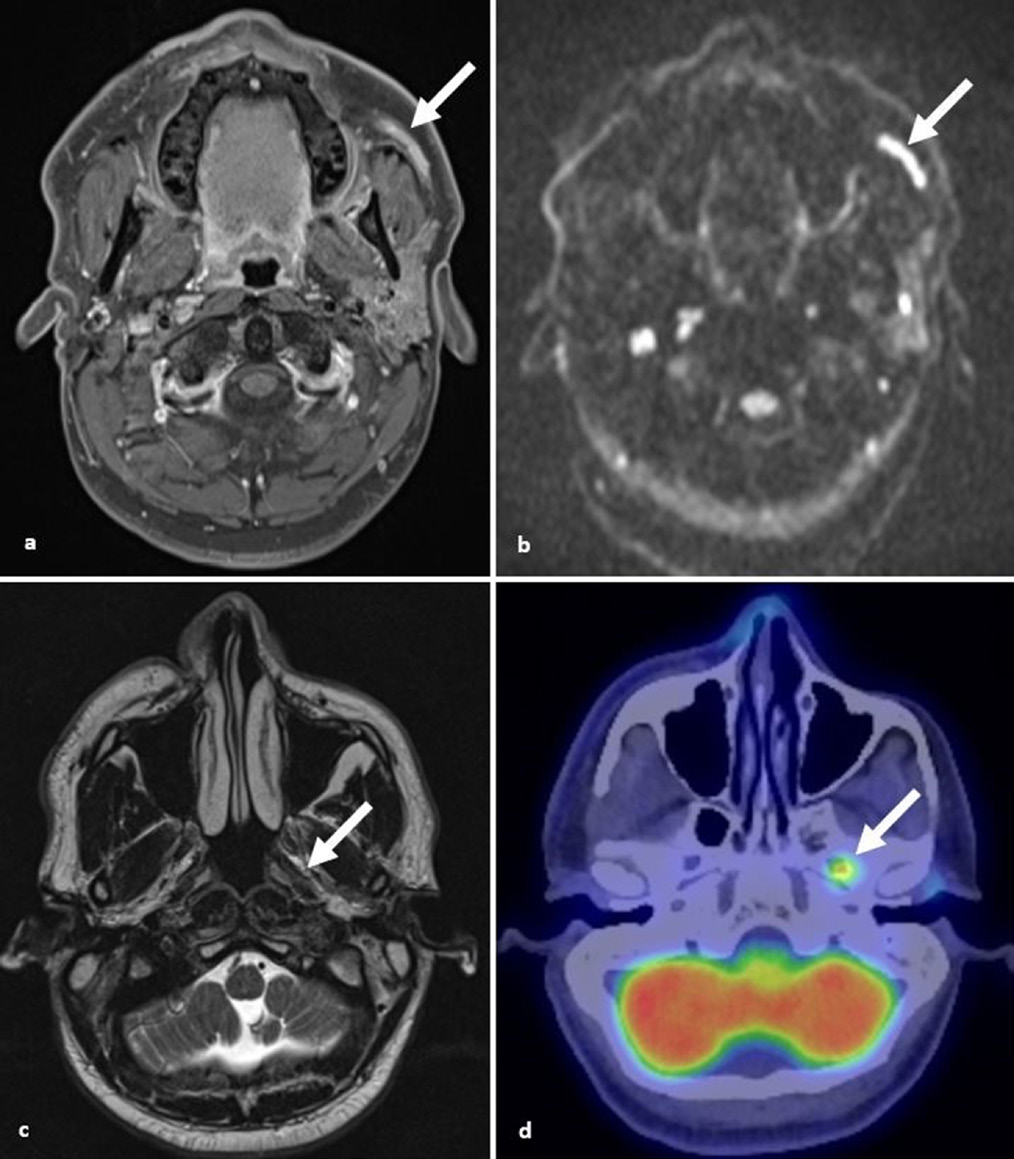
Malignant melanoma. (**a, b**) Axial T1 post-gadolinium fat saturated and DWI images demonstrate thickening and enhancement of the left buccal branch of the facial nerve with restricted diffusion (arrow) due to previous cutaneous melanoma (arrows). (**c**) Plaque of intermediate T2 signal extending within the retromandibular region representing spread along the auriculotemporal nerve (arrow). (**d**) Axial PET CT 6 months later showing uptake at the foramen ovale due to extension along the mandibular nerve (arrow). DWI, diffusion-weighted imaging; PET, positron emission tomography.

Most melanomas occur *de novo* rather than within a pre-existing naevus. On MRI, lesions typically show T1 hyperintensity and T2 signal heterogeneity secondary to either paramagnetic melanin or haemorrhage. They also demonstrate heterogenous enhancement, peritumoral fat stranding, ill-defined deep tumour margins and perineural spread (Figure 11).^9^ Positron emission tomography-CT is the preferred modality to assess for metastatic spread.

### Primary cutaneous lymphoma

Primary cutaneous lymphomas are a group of lymphoproliferative neoplasms, limited to the skin, with no involvement of lymph nodes, viscera or bone marrow at diagnosis. The majority are T-cell lymphoma (TCL) (65%), followed by B-cell lymphoma (BCL) (25%).^10^

**Figure 12.**
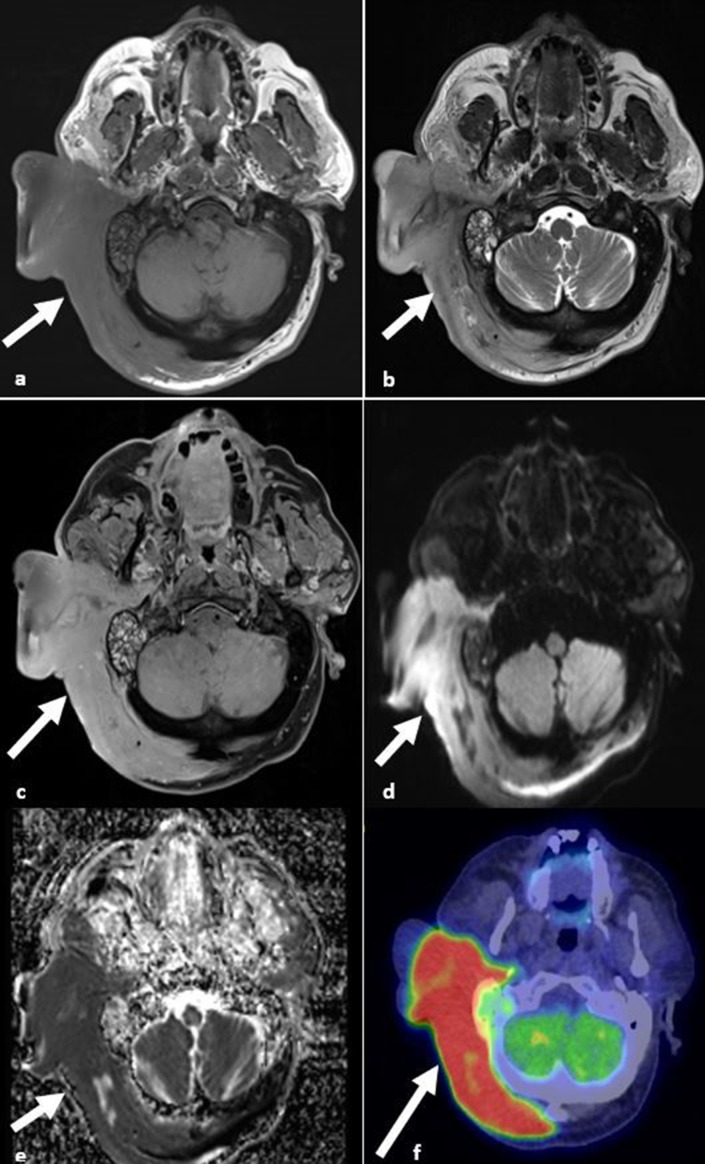
Primary cutaneous lymphoma. (**a, b**) *T*_1_W and *T*_2_W images demonstrating intermediate signal in keeping with extensive infiltration of the subcutaneous tissues and occipital and suboccipital muscles (arrows). (**c**) T1 post-contrast image demonstrates homogeneous enhancement of the lymphomatous infiltration (arrow). (**d, e**) DWI and ADC images demonstrating restricted diffusion of the lymphomatous tissue (arrow). (**f**) PET/CT image demonstrating increased avidity of the lymphomatous tissue (arrow). ADC, apparent diffusion coefficient; DWI, diffusion-weighted imaging; PET, positron emission tomography.

The initial patch, plaque or erythroderma stages are difficult to discern on imaging, however, there is subsequently a “thorny” extension to the subcutis due to lymphatic infiltration. On CT/MRI, cutaneous TCL may show diffuse skin thickening, nodularity and ulceration. Whilst more typically occurring on the trunk, they may occur in the head and neck. Cutaneous BCL often presents as an isolated nodule/mass (Figure 12), and are usually located in the head and neck. Fluorodeoxyglucose positron emission tomography/CT almost always demonstrates increased uptake (Figure 12f).^10^

### Dermatofibrosarcoma and Kaposi’s sarcoma

**Figure 13.**
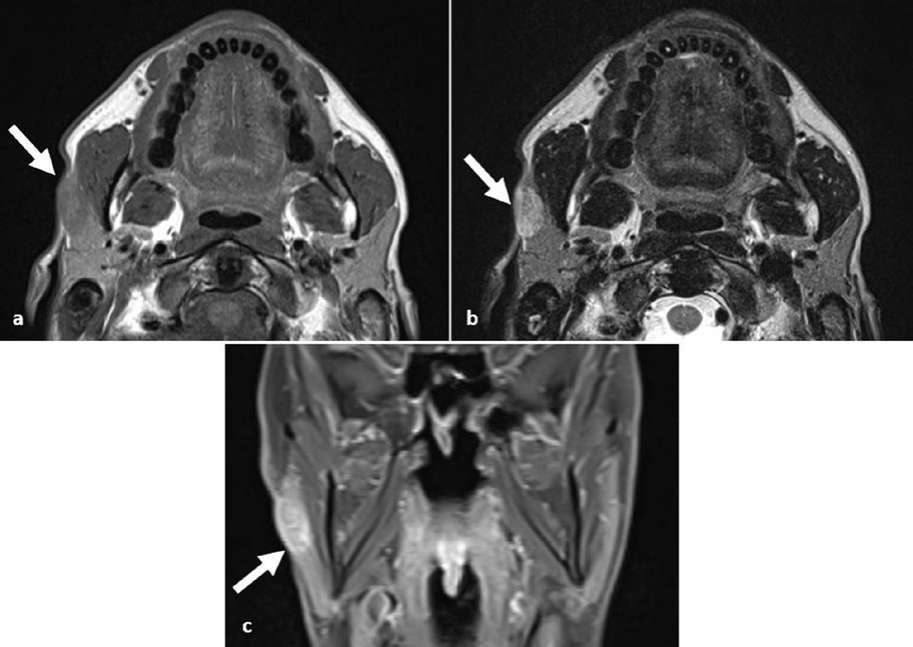
Dermatofibrosarcoma and Kaposi’s sarcoma. (a–c) Axial *T*_1_W, axial *T*_2_W and coronal post-gadolinium *T*_1_W fat-saturated images demonstrate an intermediate T1 enhancing lesion within the right pre-auricular region extending from the cutaneous surface deeply to involve the superficial lobe of the parotid gland (arrows).

Dermatofibrosarcoma is a rare, slow growing cutaneous sarcoma which develops in the dermis and typically protrudes superficially. Deeper infiltration is described involving the subcutis, and muscle (Figure 13). The head and neck is the primary site in 10–15% of cases.^11^ They typically demonstrate avid contrast enhancement and may appear heterogenous due to haemorrhage.^12^ Kaposi’s sarcoma is a further low-grade sarcoma which may be AIDS-related. It rarely involves the face but is characterised by marked enhancement.

## Blistering skin lesions

Epidermolysis bullosa (EB) is a rare genetic blistering disorder characterised by epithelial fragility, resulting in blistering (Figure 10a and b) after minor trauma. The most common cause of mortality in severe EB is SCC.^13^

## Neurocutaneous

### Sturge weber syndrome (SWS)

SWS is a rare, sporadic neurocutaneous syndrome characterised by eye, skin and meningeal angiomatosis, as well as a facial cutaneous capillary malformation (port wine stain) in an ophthalmic division of V1 nerve distribution.^14^

**Figure 14.**
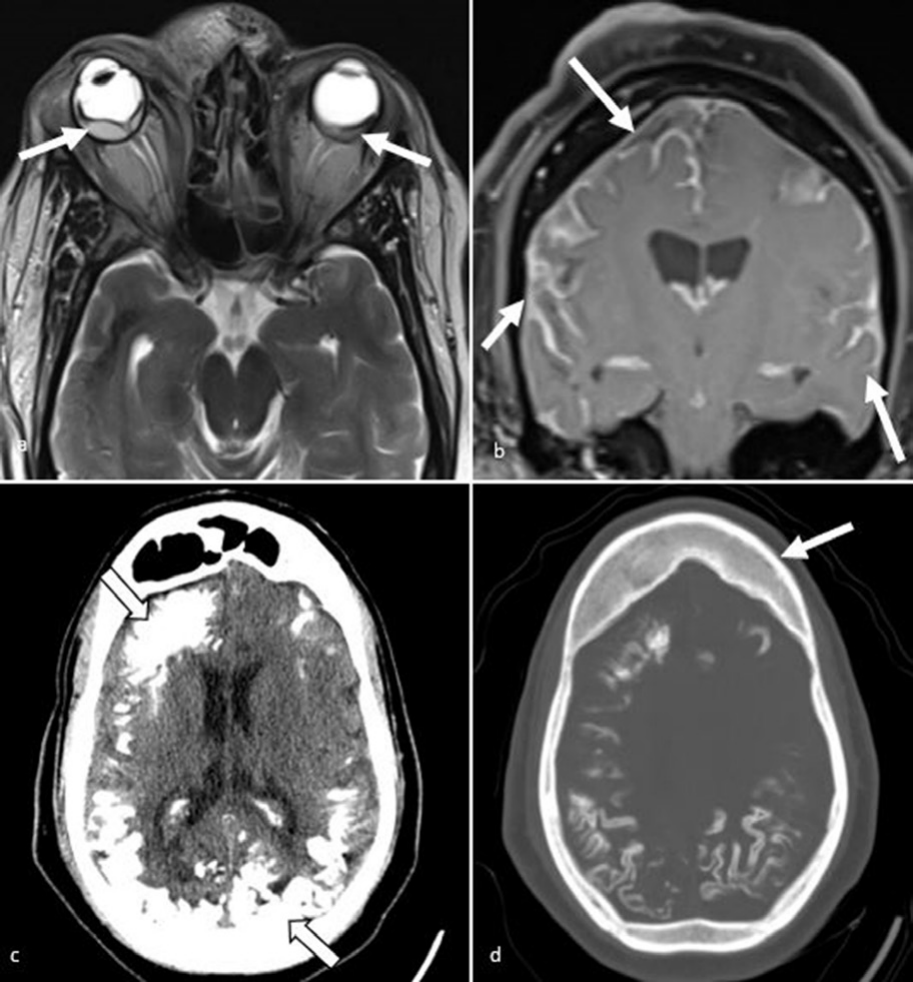
Sturge weber syndrome. (**a**) Axial *T*_2_W images demonstrate bilateral retinal detachment due to choroidal angiomas (arrows) and soft tissue thickening in the right frontonasal region. (**b**) Coronal T1 post-contrast images demonstrate diffuse bilateral pial/leptomeningeal enhancement, more apparent on the right (arrows). (**c**) Axial CT images demonstrates widespread tram track gyriform calcification (black arrows). (**d**) Axial CT images on bone windows demonstrates thickened calvarium of the frontal bones (arrow).

Imaging plays a crucial role in diagnosis and establishing intracranial ancillary features. Imaging findings (Figure 14) include cortical/subcortical calcification, calvarial enlargement, parenchymal volume loss, white matter T2 hyperintense ischaemic change, ipsilateral choroid plexus enlargement, leptomeningeal enhancement and prominent transparenchymal collateral veins.^15^

## Neurofibromatosis type 1 (NF1)

**Figure 15.**
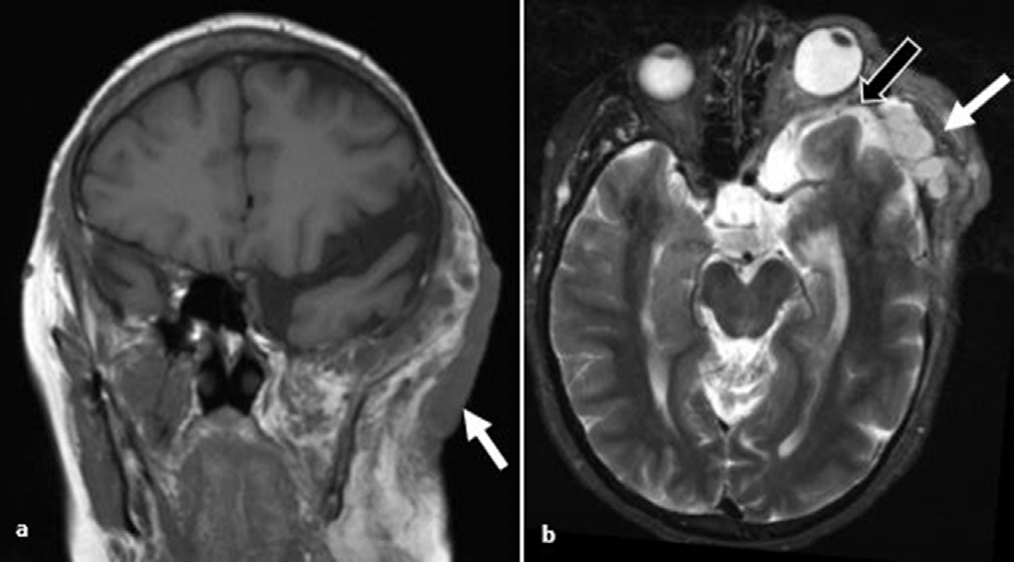
Neurofibromatosis type 1. (**a**) *T*_1_W coronal image. Diffuse superficial plexiform neurofibroma diffusely infiltrating the left buccal and temporal regions (arrow). (**b**) *T*_2_W axial image demonstrates plexiform neurofibroma within the temporal fossa (white arrow) associated with sphenoid wing dysplasia (black arrow) and marked proptosis.

NF1 is a multisystem genetic neurocutaneous disorder. Cutaneous neurofibromas may be limited to the skin. Plexiform neurofibromas are deeper lesions, however, cutaneous/subcutaneous involvement is common. Plexiform neurofibromas (Figure 15) are more strongly associated with NF1, typically unilateral or asymmetric with ill-defined margins, and increased vascularity resulting in avid enhancement.

## Conclusion

Whilst cutaneous lesions are usually diagnosed clinically or after biopsy, imaging plays a key role in assessing disease extent and associated complications. Although some imaging appearances are non-specific, those characteristics features of cutaneous lesions listed in Table 2 will aid a radiological differential diagnosis. The radiologist should recognise the characteristic imaging features of benign incidental cutaneous lesions, and in particular those that may mimic aggressive lesions. An understanding of systemic, syndromic or neurological associations will also result in an appropriate radiological search. It is hoped this review will increase awareness of these cutaneous lesions and will help the framing of a clinically relevant report.

**Table 2.** Summary of skin lesion findings and their complications.

	Key imaging features	Tips and pitfalls
**Overgrowth (increase font size)**		
Cutis VerticisGyrata	Cutaneous ridges and furrows on scalp	Consider underlying endocrinological or dermatological conditions
Giant Keloid	Exophytic T2 hypointensity, and may show areas of enhancement	Enhancing superficial mass should be correlated with clinical history to avoid misdiagnosis as an aggressive tumour
**Benign**		
Haemangioma	Avid enhancement. Flow voids.	Evaluate encroachment on vital structures (*e.g.,* orbits, and airway) since treatment with propranolol will be considered. Maybe associated with systemic hemangiomas and PHACES syndrome (posterior fossa malformations, haemangiomas, cerebral/cervical artery malformations, cardiac abnormalities / aortic coarctation, eye abnormalities).
**Skin Appendage Lesions**		
Epidermal inclusion cysts	Well defined with increased DWI signal.	Do not misinterpret restricted diffusion as indicating a cellular aggressive lesion
Hydradenitis Suppurativa	Diffuse STIR hyperintense oedema	Determine deep extent and assess for associated abscesses
Pilomatrixoma Buccal	Bands of T2 hyperintense signal radiating from a low signal centre Usually calcified	Associated with other conditions (*e.g.,* sarcoid) and syndromes. Characteristically midface location
Cylindroma	Well demarcated nodular lesions	Multiple lesions associated with Brooke-Spiegler syndrome
Nodular Hidradenoma	Complex cystic and solid	Solitary lesion with predeliction for scalp and face in adult females
**Malignant**		
Cutaneous SCC	Ill-defined tumour margins, superficial irregular ulceration.	Evaluate deep invasion, bone erosion perineural spread, lymph node and distant metastases
Malignant Melanoma	Ill-defined tumour margins. Potentially T1 hyperintensity, heterogenous T2 hypointensity.	Evaluate deep invasion, perineural spread, lymph node and distant metastases
Primary cutaneous face/ scalp lymphoma	TCL - Skin thickening, nodularity and ulceration. BCL – Isolated nodule / mass	Cutaneous BCL usually located in the head and neck
Sarcoma: Dermatofibrosarcoma and Kaposi’s sarcoma	Avidly enhancing, Dermatofibrosarcoma typically protruding outwards with haemorrhage	Kaposi’s sarcoma is AIDS related
**Blistering Skin Lesions**		
Epidermolysis Bullosa	Imaging reflects vesicular or scarring (T2 hypointensity) phases	Increased risk of skin malignancies, particularly SCC and structuring of the digestive tract due to mucous membrane involvement
**Neurocutaneous**		
Sturge Weber syndrome: Facial cutaneous capillary malformation (port wine stain)	Clinically diagnosed	Intracranial ancillary features: Cortical/subcortical calcification, calvarial thickening, dilation of transependymal veins, leptomeningeal enhancement, orbital haemangiomas. Note the facial capillary malformation and intracranial features may both occur in isolation
Neurofibromatosis Type 1: cutaneous and plexiform (deep) neurofibromas	Plexiform neurofibroma has ill-defined margins and often associated vascularity with associated bony dysplasias in the head and neck.	Multi system disorder, with cutaneous, central nervous system (*e.g.,* gliomas), musculoskeletal, pulmonary and orbital manifestations. Rapidly growing or painful plexiform neurofibromas may signify conversion to malignant peripheral nerve sheath tumour
